# Investigating the role of three screening measures to support clinical decision-making in adult autism assessments

**DOI:** 10.1371/journal.pone.0333875

**Published:** 2025-11-13

**Authors:** Janine Robinson, Carrie Allison, Bonnie Auyeung, Isabel C. H. Clare, Michael Lombardo, Navneet Nagra, Simon Baron-Cohen

**Affiliations:** 1 Cambridgeshire and Peterborough NHS Foundation Trust (CPFT), Cambridge, United Kingdom; 2 Department of Psychiatry, Autism Research Centre, University of Cambridge, United Kingdom; 3 Psychology Department, School of Philosophy, Psychology and Language Sciences, University of Edinburgh, Edinburgh, United Kingdom; 4 Department of Psychiatry, University of Cambridge, Cambridge, United Kingdom; 5 Laboratory for Autism and Neurodevelopmental Disorders, Center for Neuroscience and Cognitive Systems, Istituto Italiano di Tecnologia, Rovereto, Italy; 6 Central and North West London NHS Foundation Trust, London, United Kingdom; KI: Karolinska Institutet, SWEDEN

## Abstract

Referrals for autism diagnostic assessments in adults are increasing, with demand creating long waiting lists. Rigorously evaluated screening tools could serve to identify who is most likely to receive an autism diagnosis and contribute to clinical decision-making. We retrospectively examined individuals attending a specialist diagnostic assessment service in the UK over four years (2011–2014). Complete data on three screening measures were available for N = 422 referrals. These were the Autism Spectrum Quotient (AQ), the Empathy Quotient (EQ) and the Childhood Autism Spectrum Test–Relatives’ Questionnaire (CAST-RQ). 89% (n = 376) received an autism diagnosis. Positive screens on all three measures had a 98.3% likelihood of receiving an autism diagnosis, confirming findings from an independent clinic sample. We also examined the AQ subscale scores to establish their association with diagnostic outcome. While all people accepted onto the diagnostic assessment pathway should be offered an assessment, people who meet all three cut-offs could be offered a briefer assessment given the high likelihood that an autism diagnosis will be confirmed. Such triaging may lead to more efficient use of clinic time allowing referred individuals to be seen more quickly.

## Introduction

Autism spectrum conditions (hereafter autism) are neurodevelopmental, characterised by impairments in reciprocal social interaction and communication, alongside unusually repetitive behaviours and narrow interests, difficulties adjusting to unexpected change, and sensory hyper- or hypo-sensitivity [1]. Latest reports suggest that autism affects approximately 1 in 36 with a reported overall ratio of 4–1 in self-reported men and women, allegedly increasing in autistic people without additional intellectual disability [2–4]. The gender ratio in autism diagnoses persists even after accounting for under-diagnosis and/or misdiagnosis and camouflaging among girls and self-reported women [5,6]. Most autistic people (75%) who are diagnosed in adulthood do not have an additional intellectual disability [3,7]. Many of these individuals seek confirmation of a diagnosis after years of struggling with their daily lives and mental health. Such adults have been referred to as ‘the lost generation’ [8,9] without access to services and, in many jurisdictions, supportive legislation that might lead to alleviation of their difficulties.

Nevertheless, the past decade has seen responses internationally with the introduction of legislation, e.g., the Autism Collaboration, Accountability, Research, Education and Support (CARES) Act of 2019 in the USA [10] and the Autism (England & Wales) Act 2009 [11] in the UK. The latter requires local authorities and national health system organisations to provide clear pathways for the assessment and diagnosis of autism for adults. To support implementation of legislation in England and Wales, the revised *National Strategy for Autistic Children, Young People and Adults: 2021–2026* [12] and clinical guidelines and quality standards [13] have been produced. Elsewhere, organisations have produced guidance for assessment and diagnosis relevant to their jurisdictions: for example, in France [14] and in Australia [15].

Among individuals without additional intellectual disability, more than half of autistic adults meet criteria for depression (63%) or anxiety disorder (43%) [16], potentially as a result of late diagnosis [17]. The average age of diagnosis in adulthood is 31–49 years, for both men and women [18,19] The increasing number of referrals to adult services is likely due to greater awareness of autism within the wider public and higher levels of expertise within the health system [20]. However, increased numbers of referrals of people with complex presentations have also been reported [21], with clinicians describing concerns about their competence to diagnose subtle forms of autism, including differences in its presentation in women, masking by co-occurring conditions such as borderline personality disorder [22,23] or anorexia nervosa [24], or by ‘camouflaging’ [25]. Camouflaging refers to coping strategies employed by a person in social situations to fit in, to hide those behaviours that might be typically associated with autism and may include explicit techniques to present as socially competent. It may be more common in autistic women [25]. Furthermore, some individuals are able to employ compensatory strategies, thus presenting less overt autistic features and demonstrate, for instance, modulated eye contact and appropriate social reciprocity [6]. Complexity of presentation in adulthood requires the expertise to distinguish autism from other conditions [26,27].

Increasing numbers of adult referrals place pressure on diagnostic assessment services to meet demand. Even in countries with well-organised health care systems, waiting times remain a concern because referrals often occur when an individual is in crisis. Delays in accessing an accurate diagnostic assessment can impact on their quality of life while they wait for appropriate support and intervention dependent on the diagnosis [28,29]. In September 2024 in the UK, 204,876 people were waiting for a diagnostic assessment, representing a 25% increase in just one year in the number of people waiting [30]. In adults, waiting times for a diagnostic assessment can be long [29], which is unacceptable to individuals, their families and clinicians [31,32]. Furthermore, suicidality risk in autistic adults (including suicidal ideation and suicide plans or attempts) is higher than in the general population: Cassidy et al. [33] found a 66% lifetime experience of suicidal ideation and a 35% lifetime experience of planned or attempted suicide in adults diagnosed with autism without intellectual disability. This may be exacerbated by barriers in accessing health and social care services [34]. For many adults, confirmation of a diagnosis is necessary to access statutory services, statutory benefits, and support or intervention for additional needs.

Health practitioners need reliable screening measures to identify individuals who may require a referral to a specialist autism diagnostic assessment service. Equally, diagnostic assessment services themselves would benefit from screening measures that could help check if referrals are appropriate. Various self-report and informant-report screening measures are available for clinicians to support the adult referral pathway for a diagnostic assessment. These include the Autism Spectrum Quotient (AQ) [35,36]; the Empathy Quotient (EQ) [37]; the Autism Spectrum Disorder in Adults Screening Questionnaire (ASDASQ) [38]; the Social Communication Questionnaire (SCQ) [39]; the Social Responsiveness Scale (SRS) [40]; the Ritvo Autism Asperger Diagnostic Scale-Revised (RAADS-R) [41] and the Gilliam Autism Rating Scale-3rd Edition (GARS-3) [42]. In England, the only measure that is recommended by the National Institute for Health and Clinical Excellence (NICE) to support a referral for a specialist assessment in people who do not have an additional intellectual disability is a short form of the AQ (AQ-10) [13,43–45].

Previous studies examining the AQ and its various shorter forms (AQ-10; AQ-20 and AQ-28) have produced mixed results. Brugha et al. [2] used the AQ in a general population survey in England and found the AQ (20-item version) modestly predicted a diagnosis of autism (meeting the ADOS 10 + threshold). The AQ-20’s sensitivity in a sample of 618 adults was 0.73 and specificity was 0.62.

A further study by the same authors [46] compared the effectiveness of the RAADS-R and the AQ (50 items) in identifying autism in adults in mental health services. They reported sensitivity in a sample of 364 men and 374 women of the AQ to be 0.79 (95% CI: 0.54, 0.94) and specificity to be 0.77 (95% CI: 0.65, 0.86) at a cut-point ≥31, and sensitivity of the RAADS being 0.75 (95% CI [0.48, 0.93]) and specificity being 0.71 (95% CI [0.60, 0.81]) at cut-point ≥120. They concluded that the AQ and RAADS can guide decisions to refer adults in mental health services to autism diagnostic assessment services, but that neither of these measures can be used in isolation. Rather they should be used in conjunction with clinical judgement and other autism assessment measures. Ashwood et al. [47] investigated the AQ as a predictor of autism ‘caseness’ in a sample of 476 adults referred to a diagnostic assessment service and found sensitivity of the AQ at a cut-off total score of 26 was 0.88 (95% CI:0.84–0.91), and specificity was 0.2 (95% CI: 0.13–0.28). Using a cut-off of 32, sensitivity was 0.71 (95% CI:0.65–0.75) and specificity was 0.35 (95% CI: 0.27–0.44). Sensitivity of the short AQ (10 items) was 0.77 (95% CI: 0.72–0.82), and specificity was 0.29 (95% CI: 0.20–0.38) using a cut-off of ≥6. Scoring below the cut-off on both the AQ and AQ-10 resulted in a significant number of false negatives. The authors also included an informant-version of the AQ, but do not report on the combined predictive validity of the self-report versions with the informant-report versions; that would be useful to establish.

The Empathy Quotient (EQ) [37] is a quantitative self-report measure of empathy, namely the drive to identify another person’s thoughts and emotions, and to respond to these with an appropriate emotion. It comprises three core subscales: cognitive empathy, emotional empathy and social skills. The EQ has been found to predict scores on the AQ with lower EQ scores associated with higher AQ scores in non-autistic men [48]. In the general population the mean score for women has been reported as 47.2 (SD = 10.2) which is significantly higher than the mean score of men in the general population as 41.8 (SD = 11.6) while autistic people scored lowest at 20.4 (SD = 11.6) [48].

Developmental history from an informant is a useful adjunct to self-reporting of autistic traits at referral. A brief history related to the possible presence of autistic traits in childhood can provide key information for clinicians to guide the nature and format of the assessment. The Childhood Autism Spectrum Test – Relatives’ Questionnaire (CAST-RQ) is adapted for use with adults from the CAST [49–51], a validated parent-report autism screening instrument that has been used in epidemiological research. The CAST-RQ has not yet been validated in an adult clinical population but is used in combination with self-report measures (AQ and EQ), as an additional informant-report developmental history measure for referrals to a UK specialist autism diagnostic assessment service.

Using a combination of screening measures of current autistic traits, together with a developmental history, could streamline the pathway for adults seeking an autism assessment [52]. Previous research has examined the predictive validity of a single screening instrument (AQ) applied to referred adults [47,53,54] finding less than optimal validity. The AQ, AQ-10 and EQ are commonly used across services in England and elsewhere, but not necessarily in combination with each other or alongside a developmental measure such as the CAST-RQ. A recent clinical study [55] employed a combination of the AQ, EQ and CAST-RQ in a clinical setting. The authors found that the three screening measures had good sensitivity to predict a positive diagnosis of autism, but specificity was poor. Moreover, the predictive validity of the combination of the three measures was limited.

In light of this earlier work, the present study was a service-focused evaluation, without any a priori hypotheses. First, we investigated whether a combination of scores on three screening measures (AQ, EQ and CAST-RQ) was positively associated with an autism diagnosis in a retrospective clinic sample of people attending a specialist autism diagnostic assessment service. To do this, we examined the diagnostic outcome for referrals in relation to the scores from the three measures at the point of referral. Secondly, we examined the association of AQ subscale scores with diagnostic outcome.

## Methods

### Ethics

This study used existing clinical data that were anonymised by the lead author and did not require independent ethics review. The study was registered with the Department of Clinical Effectiveness at the local mental health provider in England (UK). Since the project was granted approval as service evaluation, it met all the necessary requirements expected by the mental health provider and no participant consent was required.

#### Study design and setting.

A retrospective case review was conducted on adults who had accessed the service and did not have an intellectual disability, as determined by self-report or informant evidence. This included factors such as absence from the General Practitioner’s Learning Disability Register, attendance at mainstream or designated Special Educational Needs (SEN) provision, or, in older individuals, a history of having been denied access to formal education.

At the time of the study, the service accepted direct referrals from primary care, mainly General Practitioners, secondary services, and higher education (college) support services. Self-referrals were not accepted. The policy of the clinic was to not accept individuals who had previously been assessed by another clinic for autism, except in exceptional circumstances, for example, where a full developmental history had not been available at the time of the first assessment. Assessments were conducted by qualified clinical psychologists and psychiatrists who had been trained and were supervised. At the time the service was established, the DSM-IV-TR [56] diagnostic terminology, including Autistic Disorder and Asperger’s Disorder, was used, but this was replaced with Autism Spectrum Disorder (ASD) in DSM-5 [1]. While assessing clinicians employed formal diagnostic terminology described in the diagnostic manuals, we refer to autism and employ identity-first language throughout this paper, as preferred by many autistic people [57]. The DSM classification system was used to ensure consistency with a large body of international research and to align with standardised assessment instruments that are primarily based on DSM conceptualisations.

#### Participants.

In total, (N = 460 adults (319 self-reported men (69.35%) and 141 women (30.65%)) were assessed by the service over a four-year period between 2011 and 2014. Completed AQs and EQs were available for 99.8% (n = 459) of the 460 participants and CAST-RQs for 91.96% (n = 423). Therefore, a total of 38 participants (8.26%) had at least one completed questionnaire missing. The smaller sample size for the completed CAST-RQ reflects difficulties in identifying an informant, sometimes because individuals did not consent to this part of the process, or relatives/friends were frail or deceased. One person with elective mutism was unable to complete the AQ or EQ, instead requiring an adapted assessment. Data from consecutive referrals with complete data from the AQ, EQ and CAST-RQ (N = 422 adults (295 men (69.90%) and 127 women (30.09%)) were included in the final study sample.

#### Referral processing.

Upon referral to the service, the clinical team conducted a brief review of all referrals to determine appropriateness. Referrals were only declined if they would have been better directed to another clinical service (e.g., where the referral indicated an intellectual disability or the individual was younger than 18 years). Referrals were not scrutinised for the likelihood of the presence or absence of autism.

#### Pre-assessment.

Once the referral was deemed appropriate (e.g., in terms of age, no intellectual disability), all individuals were asked to complete the AQ, EQ, and an informant (typically a parent, but otherwise a sibling who had known the referred individual as a child) completed the CAST-RQ. Paper copies of the questionnaires were sent to individuals in the post with an invitation to return the completed measures, with a questionnaire detailing demographic and background information in a stamped addressed envelope. Relevant clinical records from General Practitioners (primary care clinicians) and educational records were also requested.

Once returned, the service administrator entered the data into a macro-enabled spreadsheet. All additional relevant information obtained was made available to the clinical team to determine whether the individual could be added to the waiting list for a standard assessment. If there was evidence of likely complexity and/or risk, a joint or multiple appointment was arranged. Complex presentation includes, amongst other examples, history of trauma, involvement in care proceedings, adoption, significant history of alcohol or substance abuse, serious injury or illness, dual diagnosis, e.g., with anorexia nervosa and/or a personality disorder and/or visual or hearing impairments. It could also include the absence of a developmental informant, adverse childhood and/or adult experiences, mental health conditions resulting in admission to an acute or psychiatric hospital. For this study, data were not extracted about whether or not the referral was complex. At no point in the process were referrals declined or redirected to other services on the basis of scores on the AQ, EQ and CAST-RQ. Once the individual had been determined to be an appropriate referral (as described above), they were offered an assessment, regardless of their scores.

#### Screening measures.

**The Autism Spectrum Quotient (AQ)** [35]. This is a self-report measure of autistic traits in adults whose intellectual functioning is at least average. There are 50 items corresponding to five subscales, namely, Communication, Social Skill, Attention Switching, Attention to Detail and Imagination and four response options for each item: ‘definitely agree’, ‘slightly agree’, ‘slightly disagree’, and ‘definitely disagree’. Responses are scored using a binary system, where endorsement of the autistic trait (either mildly or strongly) is scored as 1, while the opposite response is scored as 0; the maximum score is 50. Higher scores suggest a greater number of autistic traits, and in the general population, 80% of autistic individuals score 32 and above [35], while a score of 26 and above has been suggested as an appropriate cut-off in a clinically referred population [36]. The AQ is very well-established and has been translated into many languages (freely downloadable at www.autismresearchcentre.com/tests) and a short AQ (ten items) has been used in large samples of over half a million people [43,58,59]. Since the participants in the current study were recruited from a clinically referred population, a score of 26 was used as the cut-off.

**The Empathy Quotient (EQ)** is a 60-item self-report measure of both cognitive and affective empathy [37]. This original version of the EQ contains 20 filler items which are not scored. Individuals are asked to respond to each item using a 4-point Likert scale (‘strongly agree’, ‘slightly agree’, ‘slightly disagree’ and ‘strongly disagree’). An empathic response is scored ‘1’ or ‘2’ depending on the strength of the response, and other responses are scored ‘0’. Scores are summed to provide a total score between 0 and 80. Lower scores indicate lower empathy. A score of 30 or less best discriminates autistic individuals from non-autistic individuals. A cut-off of 30 was used in the current study, since, in an earlier study [37], over 80% of autistic individuals scored equal to or less than 30 compared with only 12% of nonautistic individuals.

**The Childhood Autism Spectrum Test – Relatives’ Questionnaire (CAST-RQ)** is a brief version of the CAST [49] for use in adult referrals to gather developmental history from informants. A score of 15 on the CAST was demonstrated to be a good screening cut-off in epidemiological studies in a school-age sample, since it identified 87.50% of children with a high likelihood of autism [49]. The CAST was adapted by this service as the CAST-RQ, to use as an informant-report measure to provide a brief developmental history after referral, before the assessment. Items were revised to be in the past tense. Informants were instructed to recall the individual’s behaviour between the ages of four and ten years old, coinciding with the primary school years (in England and other parts of the UK). The CAST-RQ comprises 37 items, including six filler items that are not scored but which provide information on whether the individual had any developmental or language delays. Each item is scored dichotomously with a score range between 0 and 31. It is important to highlight that the CAST-RQ has not been validated. A cut-off of 15 was used in the clinic in the absence of any research data suggesting a different cut-off would be more appropriate. Sub-threshold scores were also analysed in the current study, since these were examined in the CAST validation study of primary school age children [50]. The CAST has been translated into several languages and cross-cultural studies conducted to examine the measure’s utility and ability to identify autism in children, although these studies have not yet been peer reviewed [60,61]. A preliminary study in Nigeria found the CAST, at a cut-off of 15, to have sensitivity of 70.8%, specificity of 60.4% and positive predictive validity of 52.3% [61] suggesting this cut-off is appropriate across cultures.

#### Diagnostic assessment measures.

The **Adult Asperger Assessment (AAA)** [62], subsequently renamed the **Adult Autism Assessment** is a semi-structured interview, based on the Diagnostic and Statistical Manual of Mental Disorders, 4^th^ edition (DSM-IV-TR) criteria for a diagnosis of autism [56]. A validation study examining the AAA in a clinically referred sample suggested that it was more stringent than DSM-IV-TR criteria in identifying autistic adults without an intellectual disability [62]. More than four in every five (88%, n = 37)) individuals met the DSM-IV-TR criteria compared to 80% (n = 34) who met the more conservative AAA criteria. The AAA was re-named after a decision by the service to drop the use of the term ‘Asperger Syndrome’ to reflect that in DSM-5 and ICD-11 [1,63] the respective terms Asperger’s disorder and Asperger Syndrome were discontinued.

The original electronic version of the AAA comprised four key areas based on DSM-IV-TR criteria [56] (social interaction, restricted, repetitive and stereotyped patterns of behaviour, interests and activities, verbal and non-verbal communication and imagination). The service administrator entered the AQ, EQ and CAST-RQ responses on to the AAA. The AAA includes a macro-enabled Excel spreadsheet, whereby endorsed items from the AQ and EQ automatically populated the AAA. Items that were not endorsed were not included on the AAA, nor were the CAST-RQ endorsed items. The total scores on the AQ, EQ and CAST-RQ were available for the clinician within the Excel spreadsheet. During the clinical assessment, the clinician asked the patient and informant guided questions relating to DSM-IV-TR criteria as well as questions relevant to the endorsed items from the AQ and EQ. Clinicians then manually added examples, comments, and observations made during the assessement into the spreadsheet.

#### Clinical assessment.

Specialist health practitioners in the service routinely conduct a single diagnostic assessment appointment of up to three hours with the individual and informant, using the AAA interview. Individuals and their informants were offered the opportunity to speak with clinicians separately, if preferred, at an appropriate stage of the process. Where it was not possible for the informant to attend the joint appointment, the developmental information was gathered via a telephone interview before or after meeting the referred individual. A clinical diagnostic opinion was informed by evidence gathered during administration of the AAA guided questions, along with contextual information. Diagnoses of autism were made according to DSM-IV-TR criteria, often as a result of consensus discussions with a second clinician. The service adopted an integrated approach in which self-reported biographical details, medical and social care records, educational reports, and third-party evidence accessed ahead of time, were considered alongside formal observation and clinical assessment. Additional assessments and interviews were carried out with some referred individuals. This occurred in situations where no developmental informant was available, and/or there was evidence of a complex developmental and family history or significant mental health conditions that might have made it difficult to reach a diagnostic conclusion based on a single meeting with the referred person.

### Statistical analyses

Anonymised data were analysed using IBM SPSS Statistics version 29.0 [64]. Clinical data were initially accessed by the first author, JR, from August to December 2016 as part of a clinical fellowship programme with subsequent data checks, additional data gathered and anonymised by JR and NN, both working in the clinic. For the purposes of preparation for publication, the anonymised dataset was accessed for analyses conducted during the following periods: 11–26 November 2018; 10–15 April 2019; 20–24 April 2021; 20 July – 1 August 2021; 6–15 September 2022; 12–20 January 2023; 28 April 2024; 6 May 2024; 9 May 2024; 12–17 May 2024; 20–28 May and 16–22 June 2024 respectively.

Analyses were primarily conducted on the final study sample with complete data (N = 422), but outcomes were compared with the subgroup for whom there was not a complete data set (N = 38) in order to understand potential differences between these clinical groups. Mean differences in age of participants were compared between groups (N = 422 and N = 38), using Hedge’s *g* to allow for the large difference in sample sizes. Chi-square tests were conducted to establish the association between group membership (including gender) and diagnostic outcome.

Group differences in AQ, EQ and CAST-RQ scores (in the final study sample) were examined using independent-samples t-tests, with effect sizes reported as Cohen’s *d*. A MANCOVA was conducted to assess the effect of the independent grouping variable (autistic men, non-autistic men, autistic women and non-autistic women), on screening scores, with age included as a covariate due to significant differences in mean age between groups. Post-hoc analyses, separate univariate ANOVAs and pairwise comparisons, were performed to identify the source of any significant effects. A Bonferroni correction was applied (with a *p* value set at 0.05/3 = .017) to account for multiple testing. A preliminary analysis was conducted using independent-samples t-tests to examine differences in AQ subscale scores between individuals with and without an autism diagnosis. Effect sizes were reported as Cohen’s *d*.

Chi-square tests were conducted to examine the association between meeting cut-offs on screening measures and diagnostic outcome. For each individual measure and combination of measures (AQ; EQ; CAST-RQ; AQ and CAST-RQ; EQ and CAST-RQ; AQ and EQ; and AQ) diagnostic performance was assessed in terms of sensitivity (proportion of autistic people who screen positive), specificity (proportion of non-autistic people who screen negative), positive predictive value (proportion of people who screen positive and are autistic), negative predictive value (proportion of people who screen negative and are non-autistic) and overall accuracy (the correct classification rate).

## Results

### Participant characteristics and outcome

During the study period, 459 people aged 18 years or over attended the service and with local agreement, one young person of ~16 years was also offered an assessment (N = 460). Information about ethnicity was not available because, at the time, it was not routinely collected.

Only individuals who had completed the AQ, EQ and CAST-RQ (N = 422, 91.7%) were included in the main analyses (final study group). Table 1 shows the characteristics of the sample for: age at assessment, gender and diagnostic outcome (for those with both complete and incomplete questionnaire sets).

**Table 1:** (a) Participant characteristics of final study sample (persons attending an autism assessment appointment with complete data, N = 422) and diagnostic outcome. (b). Participant characteristics of the group who were excluded from the main analysis (N = 38) (persons attending an autism assessment appointment with incomplete data) and diagnostic outcome.

(a) Participant characteristics of final study sample and diagnostic outcome				
Characteristic		N (%)	Diagnostic Outcome Autism (N) (%)	Diagnostic Outcome Not Autism (N) (%)
**Total number of assessed participants with complete data**		422 (100%)	376 (89.10%)	46 (10.90%)
**Self-reported gender**	**Men**	295 (69.90%)	262 (88.81%)	33 (11.19%)
	**Women**	127 (30.09%)	114 (89.76%)	13 (10.24%)
**Characteristic**		**Mean and SD**	**Mean and SD**	**Mean and SD**
**Age**	**Men**	32.50 (12.28)	31.82 (11.78)	37.86 (14.84)
	**Women**	30.07 (10.89)	30.04 (11.06)	30.34 (9.77)
**(b) Participant characteristics of the group who were excluded from the main analysis and diagnostic outcome**				
**Characteristic**		**N (%)**	**Diagnostic Outcome Autism (N) (%)**	**Diagnostic Outcome Not Autism (N) (%)**
**Total number of assessed participants** **with incomplete data**		38 (100%)	27 (71.05%)	11 (28.95%)
**Self-reported gender**	**Men**	24 (63.16%)	17 (70.83%)	7 (29.17%)
	**Women**	14 (36.84%)	10 (71.43%)	4 (28.57%)
**Characteristic**		**Mean and SD**	**Mean and SD**	**Mean and SD**
**Age**	**Men**	48.87 (10.34)	47.32 (10.12)	52.66 (10.62)
	**Women**	41.72 (13.77)	43.54 (15.30)	37.19 (8.98)

An independent t-test showed that there was a significant difference in age between those with complete questionnaire sets (M = 31.77, SD = 11.92) and those with incomplete questionnaire sets (M = 46.24, SD = 12.05); *t*(458)=7.16, p < 0.001, two-tailed), with a large effect size (mean difference = 14.47, 95% CI: 10.50 to 18.44, Hedges’ **g* *= 1.21. The youngest participant was 15.98 years old; the oldest was 66.59. Levene’s Test for Equality of Variances yielded a p-value of.917, therefore indicating that equal variances could be assumed. The independent t-test indicated that there was a significant difference in the age of individuals who were diagnosed (M = 31.28, SD = 11.58) and those who were not diagnosed (M = 35.73, SD = 13.92); t(420) = −2.08, p = .04, two-tailed). The magnitude of the difference in the means (mean difference = 4.45, 95% *CI*:-8.74 −.16) was small (Cohen’s *d* = 0.375).

Of the group that did not have a complete set of questionnaires (n = 38, 8.26%), n = 27 (71.05%) received a DSM-IV-TR autism diagnosis, while n = 11 (28.95%) did not. Of these 38 individuals, 24 (63.16%) were men and 14 (36.84%) were women. More than two-thirds (70.83%, n = 17) of men in this group received an autism diagnosis, whilst 71.43% (n = 10) of women received an autism diagnosis. Of the final study sample (N = 422), n = 376 (89.10%) received a DSM-IV-TR autism diagnosis whilst n = 46 (10.90%) did not. Men and women were equally likely to be diagnosed as autistic and a chi-square test of independence (with Yates’ Continuity Correction) indicated no association between gender and diagnostic outcome, *χ*^2^ (1, n = 422) =.014, p = .91, phi = .014). Fisher’s exact test was used to determine if there was a significant association between the status of completed questionnaire sets and diagnostic outcome. There was a statistically significant association between the two variables *χ*^2^ (1, n = 460) =8.86, p = .003, phi = 0.15) and the group with complete sets (n = 376, 89.10% of 422) was more likely to receive a diagnosis of autism compared to the group with incomplete sets (n = 27, 71.10% of 38). Since it is not uncommon for adult assessment services to encounter incomplete information, such as lack of informant-completed screening questionnaires, establishing likelihood of diagnostic outcome with or without such information is useful.

### Group and gender differences on the AQ, EQ and CAST-RQ

**Fig 1:**
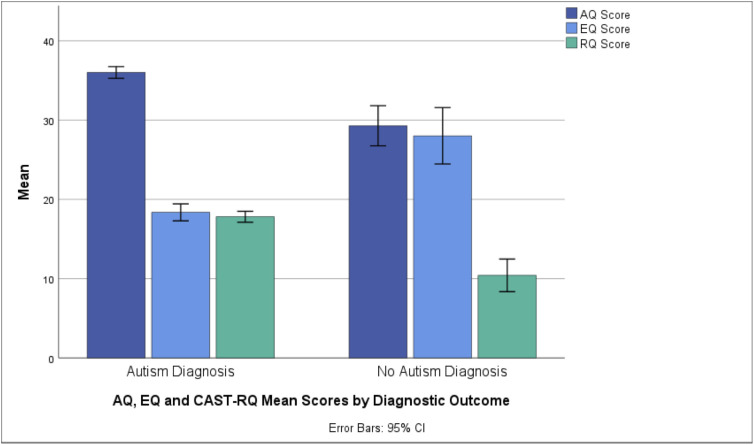
AQ, EQ and CAST-RQ mean scores by diagnostic outcome.

The mean score on the AQ for the final study sample (N = 422) was 35.28 (SD = 7.76, range: 9–49), and for the EQ the mean was 19.41 (SD = 11.15, range: 1–71). The mean score on the CAST-RQ was 17.00 (SD = 7.21; range: 0–31); see Fig 1. Higher scores on the AQ and CAST-RQ and lower scores on the EQ respectively, suggest higher autistic traits.

The group diagnosed with autism scored significantly higher on the AQ and CAST-RQ, and lower on the EQ than those who did not receive the diagnosis, with large effect sizes (0.900; 0.898 and 1.081) for the AQ, EQ and CAST-RQ respectively. Table 2 shows the summaries of independent samples t-tests for the mean scores on each of the tests by diagnostic outcome and self-reported gender.

**Table 2:** AQ, EQ & CAST-RQ group differences by diagnostic outcome for men and women.

Measure			Diagnostic Group								
			Diagnostic Outcome Autism		Diagnostic Outcome Not Autism		Total	t	df	Sig	Cohen’s *d* Effect size
			N (%)	Mean (SD)	N (%)	Mean (SD)	N (%)				
**AQ**	Self-reported gender	**Women**	114 (27.01)	37.86 (6.81)	13 (3.08)	30.69 (9.77)	127 (30.09)				
		**Men**	262 (62.09)	35.21 (7.43)	33 (7.82)	28.73 (8.09)	295 (69.91)				
	Total		376 (89.10)	36.01 (7.34)	46 (10.90)	29.28 (8.53)	422	5.76	420	<.001	.900
**EQ**	Self-reported gender	**Women**	114 (27.01)	20.05 (11.84)	13 (3.08)	31.46 (15.20)	140 (33.18)				
		**Men**	262 (62.09)	17.63 (9.94)	33 (7.82)	26.67 (10.42)	295 (69.91)				
	**Total**		376 (89.1)	18.36 (10.60)	46 (10.90)	28.02 (11.98)	422	5.75	420	<.001	.898
**CAST-RQ**	Self-reported gender	**Women**	114 (27.01)	17.47(6.42)	13 (3.08)	13.00 (7.39)	127 (30.09)				
		**Men**	262 (62.09)	17.95 (7.00)	33 (7.82)	9.39 (6.60)	295 (69.91)				
	**Total**		376 (89.10)	17.81 (6.82)	46 (10.90)	10.41 (6.94)	422	6.92	420	<.001	1.081

A MANCOVA was conducted to establish differences in scores on the three measures with respect to the independent grouping variable (autistic men, non-autistic men, autistic women and non-autistic women), There was a significant main effect of diagnostic group on scores after controlling for age*, F* (9, 1010.15) = 14.07, *p < *.001, Wilks’ Λ = .750, Partial η^2 ^= .09 (medium effect size). Separate univariate ANOVAs (adjusting for age) revealed significant main effects of group on AQ scores, *F* (3, 417) = 17.84, *p < *.001, Partial η^2^ = .114 (medium effect size), and EQ scores, *F* (3, 417) = 13.11, *p < *.001, Partial η^2^ = .086 and CAST-RQ scores, *F* (3, 417) = 15574, *p < *.001, Partial η^2^ = .101 (medium effect size). See Table 3 for pairwise comparisons. When controlling for age, women with an autism diagnosis scored significantly higher on the AQ than non-autistic men and women, and autistic men. Autistic men scored significantly higher than non-autistic men and non-autistic women on the AQ. Equally, there was a significant effect of self-reported gender on EQ scores in the autism group with autistic men scoring lower than autistic women. Autistic men and women scored statistically significantly lower on the EQ than both non-autistic men and women. Autistic men scored significantly higher than non-autistic men on the CAST-RQ and autistic women scored significantly higher on the CAST-RQ than non-autistic women. There was no significant difference in CAST-RQ scores between autistic men and women.

**Table 3:** Pairwise comparisons of AQ, EQ and CAST-RQ scores by diagnostic outcome and self-reported gender.

Comparisons							
Dependent Variable	(I) Outcome Group	(J) Outcome Group	Mean Difference (I-J)	Std. Error	Sig.^b^	95% Confidence Interval for Difference^b^	
						Lower Bound	Upper Bound
AQ score	Autistic men	Non-autistic men	7.206*	1.356	**<.001**	4.541	9.870
		Autistic women	−2.868*	.818	**<.001**	−4.475	−1.260
		Non-autistic women	4.335*	2.067	**.037**	.272	8.398
	Autistic women	Non-autistic women	7.202*	2.129	**<.001**	3.018	11.387
		Non-autistic men	10.073*	1.457	**<.001**	7.210	12.937
	Non-Autistic men	Non-autistic women	−2.871	2.392	.231	−7.573	1.831
EQ score	Autistic men	Non-autistic men	−9.120*	1.997	**<.001**	−13.045	−5.194
		Autistic women	−2.403*	1.205	**.047**	−4.771	−.036
		Non-autistic women	−13.816*	3.045	**<.001**	−19.801	−7.831
	Autistic women	Non-autistic women	−11.413*	3.136	**<.001**	−17.577	−5.248
		Non-autistic men	−6.716*	2.146	**.002**	−10.934	−2.498
	Non-Autistic men	Non-autistic women	−4.697	3.524	.183	−11.623	2.230
CAST-RQ score	Autistic men	Non-autistic men	8.175*	1.267	**<.001**	5.685	10.666
		Autistic women	.589	.764	.441	−.913	2.091
		Non-autistic women	5.044*	1.932	**.009**	1.247	8.841
	Autistic women	Non-autistic women	4.455*	1.990	**.026**	.544	8.366
		Non-autistic men	7.586*	1.361	**<.001**	4.910	10.263
	Non-Autistic men	Non-autistic women	−3.131	2.236	.162	−7.526	1.263

Based on estimated marginal means.

* The mean difference is significant at the.05 level.

^b^Adjustment for multiple comparisons: Least Significant Difference (equivalent to no adjustments).

### Preliminary analysis of AQ subscales

#### AQ subscales.

Most participants (n = 366, 86.7%) responded to all items on the AQ subscales. Four participants (0.95%) who missed more than 10% of items (≥ 6) were excluded from the analysis. Individual subscale item data were not available for a further 5 participants (1.18%) and they were also excluded from the analysis. Independent t-tests were conducted on the data from the remaining 413 participants to compare the AQ subscale scores for the groups for whom the diagnostic outcome was Autism and those for whom the outcome was Not Autism. There were statistically significant differences in mean subscale scores between the two groups with the diagnosed group scoring higher than the non-diagnosed group. The magnitude of the differences was moderate, with Cohen’s *d* for Attention Switching (0.57), Imagination (0.55), Attention to Detail (0.54) and Communication (0.67). Effect size was largest for the Social Skill Subscale (Cohen’s *d* = 0.82). See Table 4 for results.

**Table 4:** Comparison of mean AQ subscale scores for the two groups: Diagnostic outcome autism and diagnostic outcome not autism.

Subscale	Diagnostic Group						
	Diagnostic Outcome Autism	Diagnostic Outcome Not Autism	Total	t	df	Sig	Cohen’s *d* Effect size
	N = 369 (89.35%)	N = 44 (10.65%)	N = 413 (100%)				
	Mean (SD)	Mean (SD)					
**Social Skill**	7.86 (2.08)	6.11 (2.65)		4.22	49.48	<.001	**0.82**
**Attention Switching**	8.47 (1.63)	7.52 (1.97)		3.06	50.27	.004	**0.57**
**Imagination**	6.05 (2.36)	4.75 (2.28)		3.46	411	<.001	**0.55**
**Attention to Detail**	6.38 (2.38)	5.09 (2.55)		3.36	411	<.001	**0.54**
**Communication**	**7.37 (2.11)**	**5.91 (2.74)**		**4.22**	**49.27**	**.001**	**0.67**

Results from the independent t-tests showed that there were no significant differences in AQ subscale scores for men and women who did not receive a diagnosis, but diagnosed women scored on average higher than diagnosed men on the Communication Subscale, (M = 7.77, SD = 2.07) and (M = 7.20, SD = 2.11); t(367) = 2.42, p = .016, two-tailed. The magnitude of the difference in the means (mean difference = .578, 95% *CI*:.109 - 1.046) was small (Cohen’s *d* = 0.275). Diagnosed women’s mean scores on the Attention Switching Subscale was also higher (M = 8.85, SD = 1.46) than men (M = 8.31, SD = 1.68); t(237.29) = 3.12, p = .002, two-tailed). The magnitude of the difference in the means (mean difference = .541, 95% *CI*:.181−.901) was small (Cohen’s *d* = 0.335). Differences were found in mean scores on the Attention to Detail Subscale, with women scoring higher (M = 7.14, SD = 2.21) than men (M = 6.05, SD = 2.38); t(367) = 4.09, p < .001, two-tailed). The magnitude of the difference in the means (mean difference = 1.081, 95% *CI*:.561-1.601) was small (Cohen’s *d* = 0.464). No statistically significant differences were found between diagnosed men and women on Social Skill and Imagination subscales respectively.

### Screening measure cut-offs.

**Fig 2:**
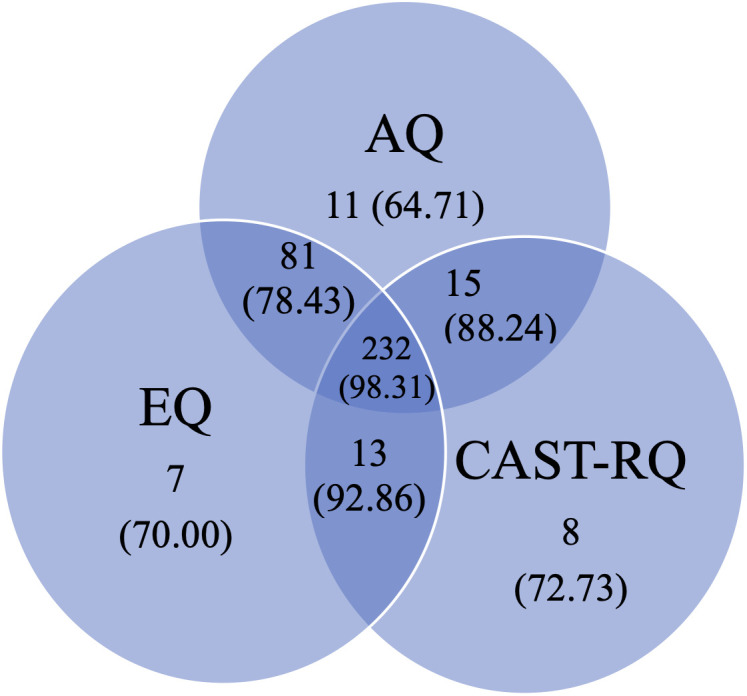
Percentage of individuals meeting cut-offs on the autism spectrum quotient (AQ), empathy quotient (EQ), CAST-relatives’ questionnaire (CAST-RQ) and combination of the measures, with diagnostic outcome autism.

**Fig 3:**
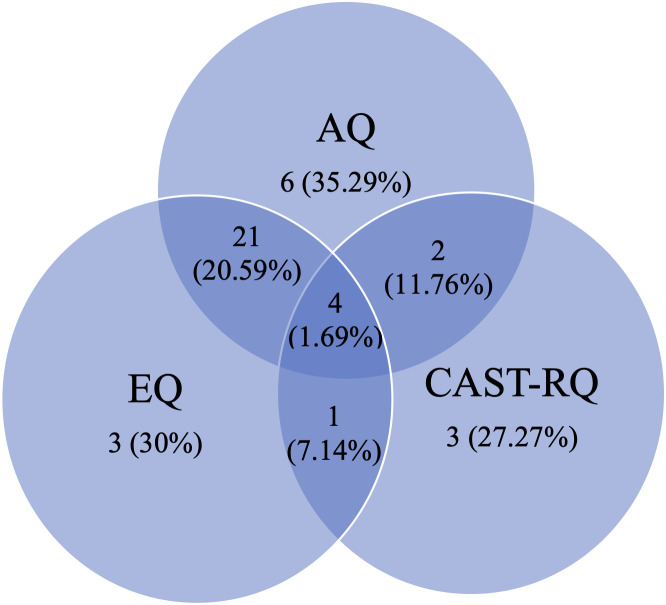
Percentage of individuals meeting cut-offs on the autism spectrum quotient (AQ), empathy quotient (EQ), CAST-relatives’ questionnaire (CAST-RQ) and combination of the measures, with diagnostic outcome not autism.

From the final study sample (N = 422), n = 38 (9.00%) participants met the cut-off on only one measure, while n = 15 (3.55%) participants did not meet the cut-off on any of the 3 measures. Of these 15 individuals, more than half (n = 9, 60.00%) nonetheless subsequently received an autism diagnosis. By contrast, half the sample (55.92%, n = 236) met all 3 cut-offs and, of these, n = 232 (98.31%) subsequently received an autism diagnosis following assessment (Table 5 and Figs 2 and 3). A chi-square test for independence was carried out for all three measures. Chi-square tests (with Yates’ Continuity Correction) indicated an association between meeting the AQ cut-off and diagnostic outcome χ^2^ (1, n = 422) = 11.61, p < .001, phi = .178 (small effect size); EQ cut-off and diagnostic outcome χ^2^ (1, n = 422) = 19.84, p < .001, phi = .23 (small effect size) and CAST-RQ cut-off and diagnostic outcome χ^2^ (1, n = 422) = 42.57, p < .001, phi = .33 (medium effect size). Provisional analysis of subthreshold scores on the CAST-RQ suggests that n = 40 (9.50%) of individuals scored between 12 and 14. Of these, n = 31 (77.50%) were subsequently diagnosed as autistic while n = 9 met the threshold but did not go on to receive a diagnosis (22.5%).

**Table 5:** Number of individuals meeting and not meeting cut-offs on AQ, EQ, CAST-RQ or combinations by diagnostic outcome.

Screening Status (+ screens positive, – screens negative)	N (%)	N (%) Diagnostic outcome Autism	N (%) Diagnostic Outcome Not Autism
AQ+ only	17 (4.03)	11 (64.71)	6 (35.29)
EQ+ only	10 (2.37)	7 (70.00)	3 (30.00)
CAST-RQ+ only	11 (2.61)	8 (72.73)	3 (27.27)
AQ + EQ+	102 (24.17)	81 (78.43)	21 (20.59)
AQ+ CAST-RQ+	17 (4.03)	15 (88.24)	2 (11.76)
EQ+ CAST-RQ+	14 (3.32)	13 (92.86)	1 (7.14)
AQ + EQ+ CAST-RQ+	236 (55.92)	232 (98.31)	4 (1.69)
AQ- EQ- CAST-RQ-	15 (3.55)	9 (60.00)	6 (40.00)

Note: The Ns are independent, rather than cumulative. Therefore, the Ns for meeting the cut-off on all three of the measures are not included in the Ns for meeting the cut-off on two of the three measures. Total N included = 422.

### Properties of the measures

The sensitivities and specificities of the three measures individually and in combination with each other are reported in Table 6. Meeting the cut-off on the combinations of the AQ and the CAST-RQ, the EQ and the CAST-RQ and the AQ, EQ and CAST-RQ respectively showed the highest specificity (0.87, 95% CI: 0.737–0.951); 0.891, 95%: CI: 0.763–0.964, and 0.913, 95% CI: 0.792–0.976) whilst the AQ had the highest sensitivity (0.902, 95% CI: 0.867–0.929). Meeting the cut-off for the EQ and the CAST-RQ, and on the AQ, EQ and CAST-RQ resulted in a 98% likelihood of receiving an autism diagnosis. Using each measure in isolation to examine the likelihood of an individual being diagnosed as autistic was less likely to predict an autism diagnosis than using a combination of measures. Investigation of sensitivity and specificity of the measures within the group that had incomplete data indicated a sensitivity of.96 for the AQ and EQ respectively. Only one person in this group had completed a CAST-RQ and subsequently met the diagnostic threshold for autism. AQ specificity was 0.09, but only one participant who was not autistic did not meet the cut-off. EQ specificity was.30. Table 6 shows individuals in the study sample who met the suggested cut-offs on each measure individually, or combination of measures. Referred individuals who did not complete all questionnaires are not reported here (n = 38, 8.26%).

**Table 6:** Total number of individuals meeting and not meeting cut-offs on each measure and combination of measures by diagnostic outcome. Sensitivity, specificity, positive predictive validity (PPV) & negative predictive validity (NPV).

Predictor	Measures completed (N)	Meeting cut-off	Diagnostic Outcome Autism (N)	Diagnostic Outcome Not Autism (N)	Sensitivity (95% CI)	Specificity (95% CI)	PPV (95% CI)	NPV (95% CI)	% Accuracy
AQ	422	372	339	33	0.902 (0.867-0.929)	0.283 (0.159-0.435)	0.913 (0.895-0.925)	0.260 (0.168-0.379)	83.41
EQ	422	362	333	29	0.886 (0.849-0.916)	0.369 (0.232-0.525)	0.92 (0.902-0.935)	0.283 (0.198-0.387)	82.94
CAST-RQ	422	278	268	10	0.713 (0.664-0.758)	0.783 (0.636-0.891)	0.964 (0.939-0.979)	0.25 (0.211-0.294)	72.04
AQ + EQ	422	339	314	25	0.835 (0.794-0.871)	0.457 (0.309-0.609)	0.926 (0.906-0.943)	0.253 (0.187-0.333)	79.38
AQ+CAST-RQ	422	251	245	6	0.652 (0.601-0.699)	0.87 (0.737-0.951)	0.976 (0.951-0.989)	0.234 (0204-0.267)	67.54
EQ+CAST-RQ	422	250	245	5	0.65 (0.601-0.699)	0.891 (0.763-0.964)	0.98 (0.955-0.991)	0.238 (0.209-0.271)	67.78
AQ + EQ+CAST-RQ	422	236	232	4	0.617 (0.566-0.666)	0.913 (0.792-0.976)	0.983 (0.958-0.993)	0.226 (0.199-0.254)	64.92

## Discussion

We investigated scores on three screening measures (the AQ, EQ and CAST-RQ) using retrospectively collected data from a sample of individuals referred to a specialist adult autism diagnostic assessment service in the UK. The aim was to determine whether the combination of self-report and informant-report developmental screening measures were associated with a diagnosis of autism in people presenting to adult autism diagnostic assessment services. In this clinical sample of 460 referred individuals who progressed to a full assessment, 88% were subsequently diagnosed with autism. Furthermore, by excluding n = 38 participants who did not provide complete datasets, this figure increased to 89.1%. More than half (55.92%, n = 236) of individuals met the cut-offs on all three measures, and of these, 98.3% (n = 232) were subsequently diagnosed as autistic.

The data suggest that the likelihood that an individual will receive an autism diagnosis following assessment is extremely high when they meet or exceed the cut-offs on all three measures. This finding has the potential for an important impact on diagnostic assessment services because scores on the three measures following referral could serve to direct the type and length of the assessment as well as the clinical expertise required, depending on the likelihood of a positive diagnosis. The combination of scores of the AQ, EQ and CAST-RQ could be used to triage patients towards a briefer assessment for those individuals who meet cut-offs on all measures, resulting in shorter wait times for diagnostic confirmation and quicker access to identification of need and appropriate support. Enhanced assessments could be offered to those who do not meet cut-offs on all measures and where complexity or atypical presentation may require fuller investigation. Such assessments may include using additional tests and gathering of information from other sources. Importantly in this study the participants who did not have complete data sets were on average older than those who had complete data sets. Additionally, men were older than women in both groups. This finding may reflect several overlapping factors. Older men may experience greater difficulty with lengthy or introspective assessments due to cognitive fatigue, age-related changes, or challenges with executive function. Cultural and generational attitudes may also play a role, as older men are often less accustomed to psychological self-report measures and may be less willing to engage fully with them. In addition, they may receive less support or encouragement to complete assessments, be less familiar with digital formats if used, or feel more ambivalent about the diagnostic process, especially if referred later in life after years of unrecognised difficulties. These factors combined could contribute to a higher rate of incomplete data in older men.

It is important to note that a proportion of individuals (3.55%, n = 15) did not meet cut-offs on any of the measures, and of these, n = 9 (60.00%) nevertheless received an autism diagnosis. Similarly, some individuals met cut-offs on only one or two measures and subsequently received an autism diagnosis. These findings highlight that these measures should not be used independently to screen patients into or away from the service, but rather as a guide to inform the appropriateness of the differentiated pathway and assessment format.

The AQ and EQ demonstrated the greatest sensitivity of the three measures (0.902 and 0.886 respectively) compared to the CAST-RQ (0.713). Specificity of the AQ was lower (0.283) than that of both the EQ (0.37) and the CAST-RQ (0.783). In contrast Bezemer et al. [65] found higher levels of specificity for the AQ in their clinical sample (0.90 at a cut-off 32 and 0.72 at clinical cut-off 26) and Jones et al. [55] found specificity of 0.50 at a cut-off 32 and 0.24 at clinical cut-off 26. It has been suggested that clinicians and researchers should examine the utility of the combined use of measures to aid the assessment, differential diagnosis and recognition of co-occurring neurodevelopmental and mental health conditions [66]. Unfortunately, in this study, we did not seek the information about co-occurring conditions that would be extremely informative in addressing this issue. To date, only one study has considered the combination of measures in the referral pathway [55].

In the original clinical validation study of the AQ, Woodbury-Smith et al. [36] found the AQ to be moderately accurate in discriminating between autistic and non-autistic individuals. The differences between the findings of these studies may reflect variations in sample size (100 *vs* 459) and/or greater heterogeneity of the samples in terms of their self-reported gender, complexity and co-occurring conditions. Furthermore, differences may also reflect the evolving understanding of autism over time, with it now being viewed as both a clinical condition and an identity [67]. The AQ in isolation performs well in case-control studies, where it discriminates autistic individuals from non-autistic individuals in the general population [44,54], but in clinical populations it is less discriminating [65]. The high level of sensitivity of the AQ in this study (0.896) suggests that it has the potential to be a useful measure to minimise false negatives; however, the low specificity suggests that in an environment of increasing pressure on services, additional information may be required to determine whether an individual should have a core diagnostic assessment. We do not suggest the AQ be used on its own to accept or decline referrals to a specialist service since it would result in assessments being offered to individuals who are not autistic. In this sample, 7.82% (n = 33) met the cut-off on the AQ but were not diagnosed as autistic. For the EQ, a further 6.87% (n = 29) met the cut-off but did not meet clinical diagnostic criteria, suggesting that the EQ also has good sensitivity but poor specificity.

Ashwood et al. [47] and Kurita & Koyama [68] propose that AQ total score may be influenced by other factors within clinical groups, such as current mental health symptoms. For example, lower AQ scores were found in autistic individuals who did not have co-occurring conditions [47]. These individuals were therefore potentially at risk of being missed. In addition, lack of awareness of, or difficulties in reflecting on, their own behaviour patterns and ‘camouflaging’ may contribute to self-reporting lower autistic traits (or higher empathy) in some individuals [25,69]. In the current study, a small number of individuals (n = 19) did not meet cut-offs on any measure, but following full clinical assessment, were diagnosed with autism. Given the reported sensitivity of the respective self-report measures, this appears consistent with expected numbers identified. However, it may be useful to employ additional measures in such instances, e.g., the Camouflaging Autistic Traits Questionnaire (CAT-Q) [69].

Currently, the cut-offs on the AQ, EQ and CAST-RQ are equivalent for self-reported men and women [35,37,70]. Studies in the general population have shown that, on average, women score lower than men on the AQ and higher on the EQ [35,37,71,72]. However, a systematic review of the AQ confirmed that these gender differences are not found within the autistic population [58]. Belcher et al. [73] reported gender bias in AQ items, whilst Murray et al. [74] found that any bias was cancelled out with men and women not scoring differently overall. Numerous studies [35,70] have reported higher rates of self-reported autistic traits in women, as was found in the current study with a mean score of 37.86 (SD = 6.81, 95% CI: 36.60–39.12) compared to a mean score of 35.21 (SD = 7.43, 95% CI: 34.30–36.11) in men. In addition, the mean AQ score in the non-autistic individuals was also higher for women. Further investigation would be useful to consider whether psychiatric symptoms might account for this pattern amongst women in a clinically referred group and whether there were simply differences in total numbers of endorsed items or differences in subscale scores of the AQ in such subgroups. Future studies should also differentiate between self-reported gender and biological sex [75].

In the current study, we primarily focused on total AQ scores but conducted preliminary analyses of the original five AQ subscale scores [35] between groups, since these may provide more useful information about group differences. Mean scores on all subscales were higher for the diagnosed group reaching statistical significance. Furthermore, the magnitude of these differences was large for the Social Skill subscale and moderate for the other four subscales. Gender differences were not observed between men and women who did not receive a diagnosis. However, within the diagnosed group women’s mean scores were higher than men’s on three subscales, particularly on the Communication and Attention Switching subscales. On the Social Skill and Imagination subscales no significant differences were found between diagnosed men and women. These relative differences in scores between men and women require further examination, in the light of reports of gender bias in screening instrument items [73].

English et al. [76] have challenged the proposed factor structure and scoring system applied to the AQ and argue for the three-factor model described by Russell-Smith et al. [77], incorporating social skills, patterns/details and communication/mindreading. They suggest that the total AQ score has a limited meaningful interpretation and argue that subscale scores should be used. Further, using a scoring system of 1–4 (rather than the original binary 0–1) with a total score range of 50–200 would yield more variance within the test. Further analysis of the current dataset might demonstrate differences in subscale score patterns between the diagnosed group who met the cut-off on the AQ and those who did not. It may be possible to identify a range, for instance, of those who are likely or very likely to meet diagnostic criteria once assessed.

Following the recommendations of an earlier study, the EQ cut-off of 30 was chosen [37]. As anticipated, autistic men and women scored lower on the EQ compared to their non-autistic peers. However, the mean scores in both the diagnosed and undiagnosed groups were below 30 (the suggested cut-off), apart from the EQ score in the undiagnosed women whose mean score was 31.46 (SD = 15.20, 95% CI: 22.28–40.65); Jones et al. [55] reported similar main group findings with the mean score for the whole undiagnosed group being 26.11 (SD = 12.3, 95% CI:13.81–38.41).

Non-autistic women had a sub-threshold mean score on the CAST-RQ of 13.00 (SD = 7.39, 95% CI: 8.53–17.47). While Jones et al. [55] did not report men and women’s scores separately, they found lower mean CAST-RQ scores for the whole group not diagnosed with autism, 13.9 (SD = 8.4, 95% CI:5.44–22.28). Investigation of an optimal cut-off for self-reported gender in boys and girls using the Hebrew version of the CAST [60] found a cut-off of 8 was better at discriminating between non-autistic and autistic girls. This was lower than the optimal cut-off for boys (13). Whilst the authors suggest future studies consider different cut-offs for self-reported gender, they also suggest further investigation into the CAST’s ability to discriminate autism from other neurodevelopmental or psychiatric conditions. These unpublished findings appear to be in contrast to the findings in our current study, where the mean CAST-RQ scores for self-reported autistic men and women were not significantly different but mean CAST-RQ scores for men who were not diagnosed as autistic were lower than for a similar group of women.

Future work should include validating the CAST-RQ as a retrospective report version of the original CAST. Further item analysis is required to establish whether higher scores might be associated with other developmental difficulties in childhood. In addition, in the context of camouflaging, it may be that informants of autistic women did not endorse items relating to autistic traits on the CAST-RQ if no educational or behavioural concerns were raised in early childhood. Therefore, it is possible that the level of parental concern in childhood may have influenced their subsequent reporting on autistic traits in childhood. No data were collected on the level of concern of the informant, so that the issue cannot be directly addressed in this dataset. Further, it will be useful to establish whether the childhood cut-off on the CAST is appropriate in the retrospective report version on the CAST-RQ, or whether different cut-offs may be more appropriate for self-reported men and women. Nonetheless, only 2.37% of this sample met the cut-off on the CAST-RQ and were not subsequently diagnosed as autistic. Provisional analysis suggests that it may be of value to consider sub-threshold scores on the CAST-RQ (scores of 12–14), since a significant number of individuals scoring in this range may meet the diagnostic criteria for autism.

Despite the limitations of the study, the findings demonstrate that meeting the cut-off on all three measures results in a 98.3% (n = 232) likelihood of being diagnosed as autistic. This means that services can be very confident that individuals who screen positive on all of these measures are highly likely to receive an autism diagnosis, creating the opportunity for services to triage these as the most appropriate referrals, and to develop a different pathway that includes a briefer assessment to confirm diagnosis for those meeting cut-offs on these three measures by a junior clinician with appropriate training and supervision. It remains important that clinicians have access to multi-disciplinary discussions, particularly where assessments prove to be more complex or require consensus decision-making. This could save the expensive cost of specialist clinicians for longer assessments of complex referrals, where there is uncertainty, or those with an unusual score presentation. Increasing interest has been expressed in the concept of stepped care and personalised health as an approach to autism [78]. Our findings support this approach, facilitating assessments that are time- and resource-efficient. Given the heterogeneity of autism, clinicians should make judgements informed by evidence regarding the most essential components of the diagnostic assessment to meet the individual’s needs. Where indicated, more detailed assessment can be conducted. This would make better use of specialists’ time and skills, allowing valuable clinical resources to be allocated to sensory profiling, and assessments of strengths and needs in the context of education, employment, health, and social care, carried out by a range of multidisciplinary professionals as part of post-diagnostic support.

To date, more work has been carried out to improve the efficiency and quality of children’s diagnostic assessment pathways [79,80] than those of adults [29]. Inefficient clinical practice has been identified as contributing to delays in accessing intervention and waiting times for children suspected of being autistic. By adopting a focused service improvement programme that includes structured collection of relevant material prior to the assessment and a tiered diagnostic assessment pathway, waiting times can be reduced and clinical time can be utilised more effectively, leading to a more cost-effective model of service provision. The current study confirms the potential value of using measures for comprehensive triaging of adult referrals to best inform decision-making around the clinical assessment of possible autism. Future studies are needed to establish whether those meeting combined cut-offs and triaged towards a briefer assessment do meet diagnostic threshold for autism and whether assessments could be completed within a brief protocol. Meeting cut-offs might strongly indicate a likely autism diagnosis but does not necessarily indicate lack of complexity.

It is important to note, there may be differences in implications of these results for different health service systems outside of the UK. In poorly-resourced countries, adult diagnosis does not lead to a clear path towards support, and therefore delayed diagnosis may further exacerbate poor mental and physical health where it is much needed. Irrespective of geographical location, there should be easily available diagnostic and post-diagnostic support services, with clinicians linking with local services to ensure that autistic people have access to educational, employment and housing support as well as having their physical and mental health needs assessed. This is a challenging task, even in well-resourced countries, because, despite improvements in childhood diagnostic assessment services, there are still significant (and increasing) numbers of adults who are undiagnosed, with very long waiting lists for assessment. Nonetheless, and in the context of increasing demand and varying levels of resources, clinicians, researchers, autistic people and families of autistic people should work together to establish what is considered to be a ‘good enough’ assessment. This is where the current study’s findings may be of value.

## Limitations

There are several limitations to this study. First, once the completed AQ and the EQ were scored, the endorsed items were visible on the Adult Asperger Assessment (AAA) that informed the diagnostic opinion of the clinician. The AAA employs a semi-structured interview format, with endorsed items on the AQ and EQ populating the interview schedule as probes for the clinician to enquire about and ascertain concrete examples of behaviour and to examine discrepancies in scoring patterns. Total scores on the AQ and EQ are provided on the front of the AAA schedule. There was also no independent evaluation to establish the validity and reliability of the diagnostic outcome: this was a convenience sample, and the study was exploratory. However, the final clinical diagnosis was based on DSM criteria, not AAA outcome, in this service, and DSM criteria do not include the AQ and EQ. A study is ongoing which is examining the AQ, EQ and CAST-RQ following referral in three clinical settings using independent diagnostic methods (i.e., not using the AAA), with the clinician being blind to these scores at assessment.

Second, and related to the point above, clinicians were not blind to the AQ, EQ and CAST-RQ scores of individuals attending assessment which could bias the diagnostic outcome. However, it is usual service protocol – and in fact recommended – for the clinician to have sight of all relevant information about the individual prior to the assessment so that they can employ an integrated approach, evaluating the individual’s background, developmental history, third-party evidence, along with self-report, observation, and clinical assessment. Furthermore, no referral was declined on the basis of scores on the AQ, EQ, or CAST-RQ, as is evident in the 17 individuals who did not meet any cut-offs and were nonetheless offered assessment appointments. Likewise, high AQ and CAST-RQ scores and low EQ scores are not treated as definitive of an autism diagnosis since experienced clinicians rightly regard the measures as self-report tools that need corroboration through examples during the clinical assessment. Indeed, in cases where scores on all the instruments reached or exceeded the cut-off, n = 4 did not go on to receive a diagnosis. This suggests clinicians are not unduly biased by any individual measure. Nevertheless, a multi-site study will address the issue, with clinicians employing independent assessment protocols (not AAA) and who are blind to the AQ, EQ and CAST-RQ scores and endorsed items.

Third, research on the AQ has highlighted possible overlap of symptom presentation between autistic individuals and those with psychiatric conditions, such as schizophrenia, Obsessive Compulsive Disorder and Social Anxiety Disorder [47,68,81]. Unfortunately, no information relating to co-occurring or differential diagnoses was available at the time of this study. This is, however, being addressed in our current multi-site study described above. Future research will investigate the impact of co-occurring conditions in autism on scores on the AQ, EQ and CAST-RQ.

Fourth, whilst preliminary analysis of the AQ subscales suggests potential value in reporting on subscale scores of individuals referred for an autism diagnostic assessment, these results are currently limited since the original data were collected some time ago during routine clinical practice when the clinic adopted the practice of binary scoring of AQ items, and exhaustive exploration of the data was not possible at this time.

Fifth, in this service sample, more than four in every five individuals who were assessed received a diagnosis of autism, suggesting a very high prevalence of autism in this population of referrals. The positive predictive value (PPV) will be high when there are many cases in the population to detect. The high prevalence of autism in this population of referrals may reflect that this clinic is mature (it is 25 years old) so that referring clinicians now have good knowledge of which individuals are appropriate to refer. Alternatively, it could be that this particular service has a lower threshold for reaching a diagnostic outcome of autism compared to other comparable services. The proportion of people diagnosed as autistic in the clinical settings noted above [47,65] (68%, versus 89% in our study), may indicate the variation of the different presentations of autism and/or that those assessed as autistic may vary as a function of clinical experience, theoretical model adopted, clinical measures and nature of disciplines involved in assessments and reaching the diagnostic opinion. This highlights the need for research which examines the diagnostic agreement between clinicians working in different services. The threshold for classifying people as autistic may differ according to practices within each service. For example, individuals may be considered to present with subclinical presentations by some clinicians yet may be considered to be clinically distinguishable as autistic by clinicians in other assessment clinics. It will be important to see whether the likelihood of receiving an autism diagnosis in those who score at or above the cut-off on all three measures is as high as the 98.3% in samples where the proportion of individuals receiving an autism diagnosis at assessment is lower than in the current sample.

Lastly, this clinical sample comprised several people with complex histories or presentations, but we were unable to delineate these from the main sample to determine whether or not the instruments performed differently in these subsets. For example, individuals experiencing substance abuse and those with a history of childhood abuse both represent complexity, but in very different ways. It will be important in future work to establish whether additional assessments are required to inform diagnosis in these individuals.

## Conclusions and future directions

We conclude that screening positive on all three screening measures offers greater utility for autism diagnostic assessment services than relying on any single measure alone. However, where services may wish to reduce burden on patients, our results support the value of the combination of one self-report measure (AQ or EQ) with an informant-report developmental questionnaire (CAST-RQ) where possible, since this combination yields good results with respect to a positive association with diagnostic outcome. In the absence of any optimal individual screening measures as concluded by Wigham et al. [52] we suggest that clinics could triage referrals and offer brief confirmatory diagnostic assessments for those referrals where cut-offs are met on a combination of screeners. Such an approach might lead to shorter waiting lists, allowing enhanced assessments to be carried out for unclear or complex referrals with unusual score presentations. Future research should confirm these results in other services using different diagnostic methods. Nonetheless, as noted by the variable outcomes across clinical services, an independent process of consensus diagnosis might be the most appropriate way to address the assessment bias of individual teams where autism diagnostic thresholds might vary substantially. The current study employed the AAA assessment tool which was based on the DSM-IV-TR diagnostic criteria for autism. Whilst an earlier study found the AAA to be more stringent than the DSM-IV-TR, examination of the association of the three measures with diagnostic outcome according to the DSM-5 and ICD-11 would be advised to establish utility within the current diagnostic systems. Investigation of possible factors impacting on scores on all measures should be studied, including co-occurring psychiatric symptoms in those with an autism diagnosis and other diagnosed or undiagnosed conditions in those who were not diagnosed with autism.

The preliminary AQ subscale results suggest that future work should focus on the expanded scoring system and explore the factor structure of the AQ to establish how the subscales should be derived in order to be most helpful. Further examination of the clinical feasibility of the 10-item version of the AQ may also be of value to reduce the burden on individuals seeking assessments.

Future research should also examine whether short forms of the three measures leads to more efficient triaging. Furthermore, evidence is required to determine what should be the core elements of a brief assessment that ensures a high-quality diagnostic experience alongside an accurate outcome. Autistic adults are a group of people whose lives are often limited when they do not have access to diagnosis and then appropriate post-diagnosis support.
